# Effects of hormonal contraception on vocal patterns of captive southern yellow-cheeked gibbons (*Nomascus gabriellae*)

**DOI:** 10.3389/fvets.2025.1574926

**Published:** 2025-04-07

**Authors:** Michal Hradec, Petra Bolechová, Hana Vostrá-Vydrová

**Affiliations:** Department of Ethology and Companion Animal Science, Faculty of Agrobiology, Food and Natural Resources, Czech University of Life Sciences Prague, Prague, Czechia

**Keywords:** gibbon, breeding programs, vocalization, zoo, *Nomascus*

## Abstract

The effects of hormonal contraception in non-human primates have been studied predominantly in relation to reproductive physiology. To date, no study has investigated how hormonal contraception affects vocal patterns in non-human primates. As part of our long-term research into the vocal behavior of southern yellow-cheeked gibbons (*Nomascus gabriellae*) in zoos, we have managed to obtain vocal datasets from four adult contracepted (Nexplanon^®^ and Depo-Provera^®^) females of this species. In addition, we also recorded coda vocalizations (i.e., male calls added after the completion of the great call) from three paired males who are partners of three of the four females studied. We quantified 11 acoustic features in the female great calls and five acoustic features in the male coda vocalizations, for which we applied a Principal Component Analysis (PCA) and subsequently components were tested using multivariate Generalized Linear Mixed Models (GLMM). Our study revealed that hormonal contraception did indeed affect the vocal structure of great call in southern yellow-cheeked gibbon females. In contrast, our study did not reveal any flexible adjustment of the structure of the coda vocalization in direct response to changes in the females’ song. In female great call, we found that the Group 1 call component and Group 2 call component were not affected by the hormonal contraceptive (Depo-Provera^®^) in the during-application period. However, it was noteworthy that once the effects of contraception had worn off (post-application period), the values of components did not return to pre-application periods but continued to change. Conversely, although the values of the Group 1 call component and Group 2 call component were most greatly affected by the contraceptive Nexplanon^®^ (during-application period). The values of both components tended to return to pre-treatment levels once the effects had waned. There was a change in the values of the Group 3 call component only after application of the contraceptive Nexplanon^®^. These values remained significantly higher than the values at the pre-application level once the effects waned. This study provides the first evidence of changes in the stable vocal patterns of female southern yellow-cheeked gibbons as a consequence of the application of hormonal contraception.

## 1 Introduction

Hormonal contraception in non-human primates is the most commonly used reproductive management tool in zoos, with the aim of eliminating problems associated with population control, such as the inclusion of surplus individuals and over-representation of offspring from certain mating pairs, or slowing rapid population growth caused by the earlier attainment of adulthood (1, 2). This should result in maintaining high welfare standards and ensuring the long-term survival of a viable non-human primate population (3, 4). In European zoos, one of the common contraceptive methods used in non-human primates is medroxyprogesterone acetate (MPA) injections (Depo-Provera®) and implants containing etonogestrel (Nexplanon®). These hormonal contraceptives, which are based on synthetic progestins, work by suppressing ovulation to prevent pregnancy (5).

Due to the phylogenetic proximity between humans and non-human primates, human hormonal contraceptives are recommended for reproductive management in captive non-human primate breeding programs (2, 4, 5). However, these of hormonal contraceptives are designed exclusively for use by women. Although the effects of hormonal contraception on reproductive systems have been relatively well documented in a range of non-human primates, from prosimians to great apes (5–16), it is surprising that little attention has been paid to its effects on behavior or communication (17–21). To our knowledge, no information regarding the effects of hormonal contraception on vocalizations in non-human primates has been published. This is despite the fact that recent studies of women using hormonal contraception have shown changes in their voices associated with the production of significantly lower F_0min_ (minimum values of fundamental frequency) and HNR (harmonics-to-noise ratio) (22–24).

To date, it is still unclear whether there are any changes in the vocal patterns of female non-human primates as a result of the use of human hormonal contraceptives.

**Figure 1:**
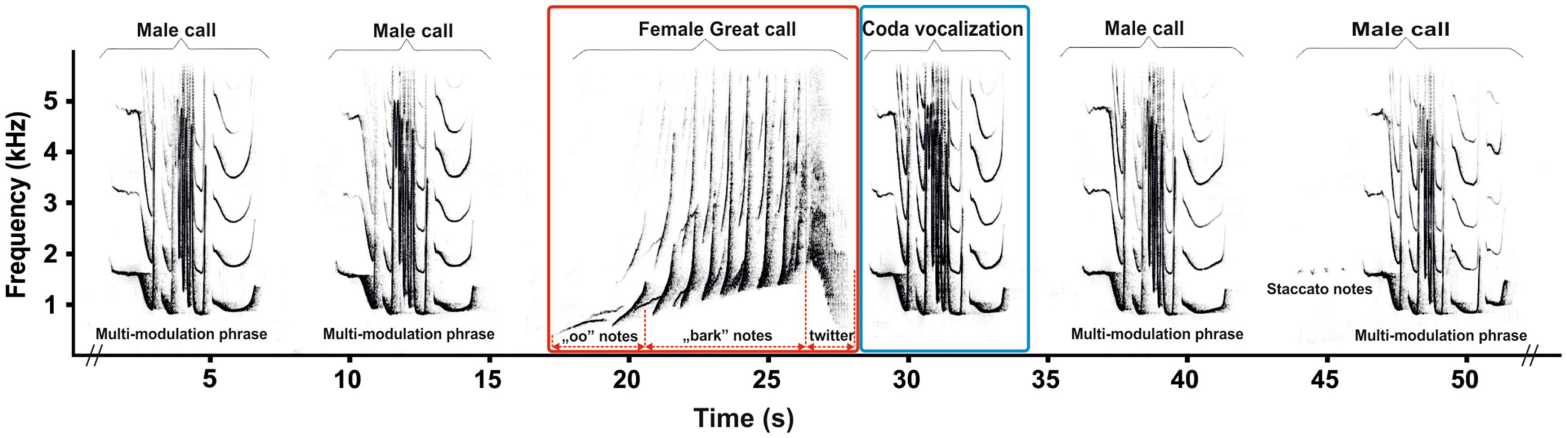
Representative spectrogram showing a duet song in southern yellow-cheeked gibbons (*Nomascus gabriellae*). During the build-up phase of the female great call (red square) in duet song, the paired male ceases his song (i.e., male call) and, after the completion of the great call, adds a coda vocalization (blue square).

Among non-human primates, gibbons (family Hylobatidae) are a relatively small, uniform group of territorial, arboreal and pair-living apes (25). Unlike great apes, however, all gibbons are well known for emitting a loud and stable pattern of vocalizations, including duets and solo songs, which are innate and specific to species and sex (25, 26). To study the effects of contraception on vocalization, adult female southern yellow-cheeked gibbons (*Nomascus gabriellae*) are a suitable model because they emit a single stable vocal pattern known as a “great call” (Figure 1, red square), which is not uttered by males (27). The complete structure of the adult female’s great call is typically an approximately 10-s phrase of 5–13 notes, and is composed of so-called “oo” sounds, “barks,” and a regularly included twitter sound. The notes produced by adult females are the dominant part of the great call, with a steep frequency increase up to 4 kHz (26, 27), and their production might be physically demanding (28). A typical male–female duet of southern yellow-cheeked gibbons begins with a few introductory notes from the male, followed by the female great call (Figure 1, red square). During the build-up phase of the female great call in a duet, the paired male ceases his song (i.e., male call) and, after the completion of the great call, adds a coda vocalization (Figure 1, blue square). Subsequently, the paired male repeats several male calls (i.e., staccato notes and a multi-modulation phrase) until the female begins her next great call (25, 27). The coda vocalization has a more routine structure than the multi-modulation phrase of the male call, and it is produced only by paired males immediately after the female great call (29). This means that the coda vocalization might be directly responding to the structure of the great call, as has been demonstrated in wild paired males of white-handed gibbons [*Hylobates lar*, (30)].

To mitigate the negative factors in captive gibbons associated with the production of surplus males and the over-representation of offspring from certain mating pairs, the EEP (European Association of Zoos and Aquaria Ex-situ Program) recommends hormonal contraception in selected females (31). This allowed us to acquire the vocal datasets of contraceptive calls from four adult female southern yellow-cheeked gibbons to investigate the effect of hormonal contraception on the structure of female great calls. To do this, we analyzed 6 years of available data from a long-term study of vocalization in these four captive southern yellow-cheeked gibbons females. In addition, we also, during this period, recorded coda vocalizations from three paired males who are partners of three of the four females studied.

This study therefore provides the first unique record of the effects of hormonal contraception on the vocal structure of the great call of female southern yellow-cheeked gibbons in three different periods (pre-application, during-application and post-application of the contraceptive). Moreover, this study also shows the response of paired males in their coda vocalization to the great call within these three periods. The aim of this study was to investigate whether the great call of female southern yellow-cheeked gibbons changes while the animal is on hormonal contraceptives and during the period when the contraceptives are expected to have worn off. A further aim was to investigate whether paired males adjust to this potential change in the structure of their coda vocalization.

## 2 Materials and methods

### 2.1 The animals used in this study

This study was conducted in Czech (Jihlava, Olomouc) and Slovak (Bojnice) zoological parks and involved four adult female southern yellow-cheeked gibbons aged between 15.6 and 38 years and their male males aged between 12 and 32 years (Table 1). Three of the four subjects lived in family groups consisting of their offspring and an adult male. Coda vocalization was recorded for these three males. One of the four females and two of the three males were born in the wild (Vietnam) and transported as a juveniles to European zoological parks in the early 1980s. The other subjects (females and males) were born in captivity.

**Table 1:** An overview of data collected from females and their mate males southern yellow-cheeked gibbons (*Nomascus gabriellae*) during three periods of hormonal contraceptive treatment (pre-, during- and post-treatment).

Zoo and Country	Number of the female; date of birth in European studbook 2022; age during study	Information about family groups (name, sex, number) in European studbook 2022 and age classes as defined by Reichard (44)	Hormonal contraceptive treatment in females		Total number of great calls and coda vocalizations recorded and days collection in “pre-application “period (2014–2015)	Total number of great calls and coda vocalizations recorded and days collection in “during-application” period (2015–2017)	Total number of great calls and coda vocalizations recorded and days collection in “post-application “period (2019–2022)
			Name and method of application	Date of application and hormone concentrations			
Jihlava (Czech Republic)	Female 1 No. 20; ~ 1984 in the wild; ~30- ~ 38 years	Adult Ad, F, No. 20 Offspring Sub/Ad, M, No. 142 Sub, M, No. 158	Depo-Provera®; intramuscularly (application every 6 months)	8 October 2015, 10 April and 8 October 2016, 9 April and 10 October 2017, 50 mg (medroxiprogesteron acetate)	Great calls: 118; 12	Great calls: 25; 4	Great calls: 21; 5
	Female 2 No. 84; 16 August 1999; 14.7–20.11 years Male 2 No. 36; ~ 1988 in the wild; 26–29; 31–32 years	Adults Ad, M, No. 36 Ad, F, No. 84 Offspring Juv/Adol, M, No. 173 Inf/Juv, F, No. 192			Great calls: 63; 7 Coda vocalizations: 52;6	Great calls: 29; 2 Coda vocalizations:29; 2	Great calls: 18; 4 Coda vocalizations: 9; 2
Olomouc (Czech Republic)	Female 3 No. 110; 9 July 2003; 10.8–18.12 years Male 3 No. 100; 22 February 2002; 12–24.4 years	Adults Ad, M, No. 100 Ad, F, No. 110 Offspring Adol/Sub, M, No. 153 Juv/Adol, M, No. 179 Inf/Juv, F, No. 204	Nexplanon®; implant (inner part of the left arm)	25 June 2015, 34 mg (etonogestrel, 1/2 dose size)	Great calls: 139; 17 Coda vocalizations:90; 14	Great calls: 96; 7 Coda vocalizations: 54;5	Great calls: 47; 5 Coda vocalizations: 13; 3
Bojnice (Slovak Republic)	Female 4 No. 80; 27 March 1999; 15.6–21.5 years Male 4 No. 37; ~ 1988 in the wild; 26, 29 and 32 years	Adults Ad, M, No. 37 Ad, F, No. 80 Offspring Sub, M, No. 148 Adol/Sub, F, No. 162 Inf/Juv, M, No. 184	Nexplanon®; implant (inner part of the left arm)	10 July 2015, 68 mg (etonogestrel, 1 dose size)	Great calls: 44; 5 Coda vocalizations: 14; 2	Great calls: 39; 4 Coda vocalizations: 27; 3	Great calls: 21; 3 Coda vocalizations: 21;3

Inf, Infants; Juv, Juvenile; Adol, Adolescent; Sub, Subadult; Ad, Adult; M, Male; F, Female.

Each group had permanent access to an indoor and outdoor enclosure featuring platforms at different heights, trees and extensive rope systems. The outdoor enclosures at two zoos (Jihlava and Bojnice) were covered with wire mesh, while the enclosure in Olomouc was equipped with a glass barrier. The gibbons were fed four times a day and provided with water *ad libitum*. Their diet consisted of fruit and vegetables, pellets for primates, browse, cereals (grain flakes), sprouted and cooked legumes, and the occasional chicken egg. At the Jihlava and Olomouc zoos, two groups of gibbons remained in visual and auditory contact.

### 2.2 Implants and sampling periods

**Figure 2:**
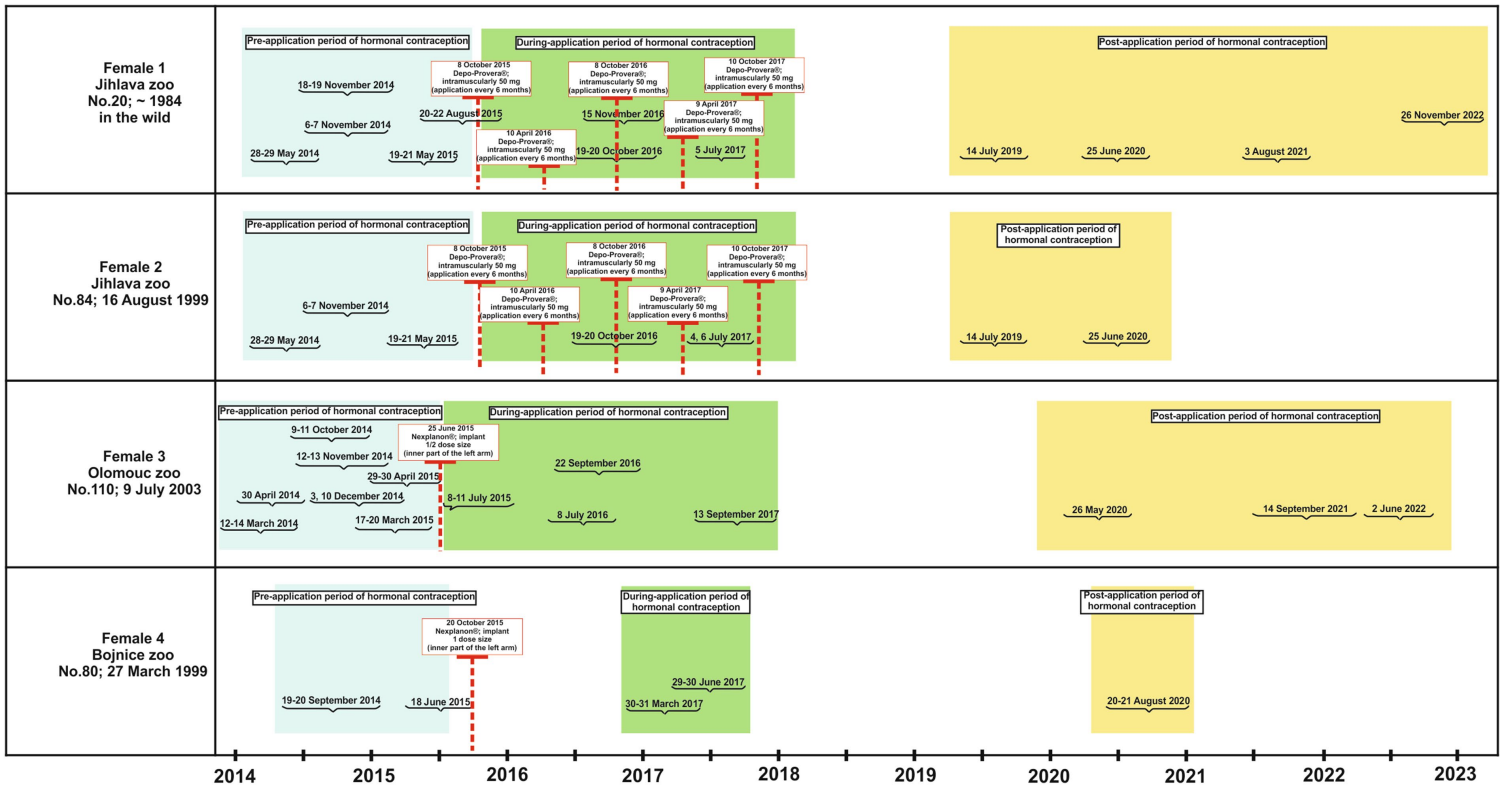
Data collected from female gibbons during three periods of hormonal contraceptive treatment (pre-treatment, during-treatment and post-treatment) including specific dates when hormonal contraceptives were administered.

Studies in a variety of non-human primate species, from prosimians to great apes, recommend injecting Depo-Provera® every 30 to 90 days, with contraceptive efficacy lasting 30 to 98 days after the last injection (5, 32). Although Depo-Provera® is designed to be well tolerated, the administration of this contraceptive in our study was conducted after a six-month (180-day) period at the discretion of the attending veterinarian. This decision was made in light of the potential occurrence of adverse effects, including liver disease, weight gain, and an increased tendency to develop *diabetes mellitus* (4). Nexplanon® is inserted subcutaneously into the (inner) upper arm and is effective for 3 to 4 years. This product is designed to be fully reversible (5). Figure 2 provides detailed information on the use of hormonal contraceptives in individual zoos. In this study, we have defined three periods of data collection as follows: the “pre-application period” is defined as any period prior to the application of contraceptives; the “during-application period” is defined as the period in which contraceptives are administered in the form of a single dose of Nexplanon® (1/2 dose at the Olomouc Zoo; full dose at the Bojnice Zoo) or repeated doses of Depo-Provera®, which are given at 6 month intervals (Table 1; Figure 2); the “post-application period” is defined as the period during which the ability to reproduce returns to its original state, prior to the application of contraceptives (reversibility). Although this process varies among species and individuals in female non-human primates (5), a study has shown that the first pregnancy in a female chimpanzee (*Pan troglodytes*) can occur as early as 2 months after the last application (8). Gibbons, as representatives of the ape group, should have an average time to reversal of 3 months, as has been suggested for female chimpanzees (5). For our purposes, we assumed the reversibility of Depo-Provera® to be 3 months after the final application. Reversibility with Nexplanon®, however, takes approximately 3–4 years (5, 31).

### 2.3 Data collection and acoustic analysis

Male and female southern yellow-cheeked gibbons produce a vocal pattern within duet or solo vocalization each of which is not normally produced by adult conspecifics of the opposite sex and exhibit no overlap in their phrase repertoires (27). It was therefore possible to identify each subject individually. Spontaneous vocalizations are typically produced every day in the morning and last approximately 15 to 30 min. Data on the great calls of the four females and coda vocalization of the three males were collected during three periods of application (pre-, during- and post-application), which took place from 2014 to 2022 (Table 1; Figure 2). Females and males were monitored for 1–4 days during each period, and the great calls and coda vocalizations were recorded from 5:00 a.m. to 12:30 p.m., typically at distances of 2 to 15 meters in the outdoor and indoor enclosures. The gibbons did not emit this vocalization after the end of the duet or solo singing. Gibbons usually keep to their morning vocalization time and therefore all vocalizations were easily recorded. We recorded 666 great calls in females and 500 coda vocalizations in males. In the females, we used all the recordings of the great call. In contrast, from the pool of recordings of the males, we extracted only 299 coda vocalizations with good quality and suitable for further analysis.

**Figure 3:**
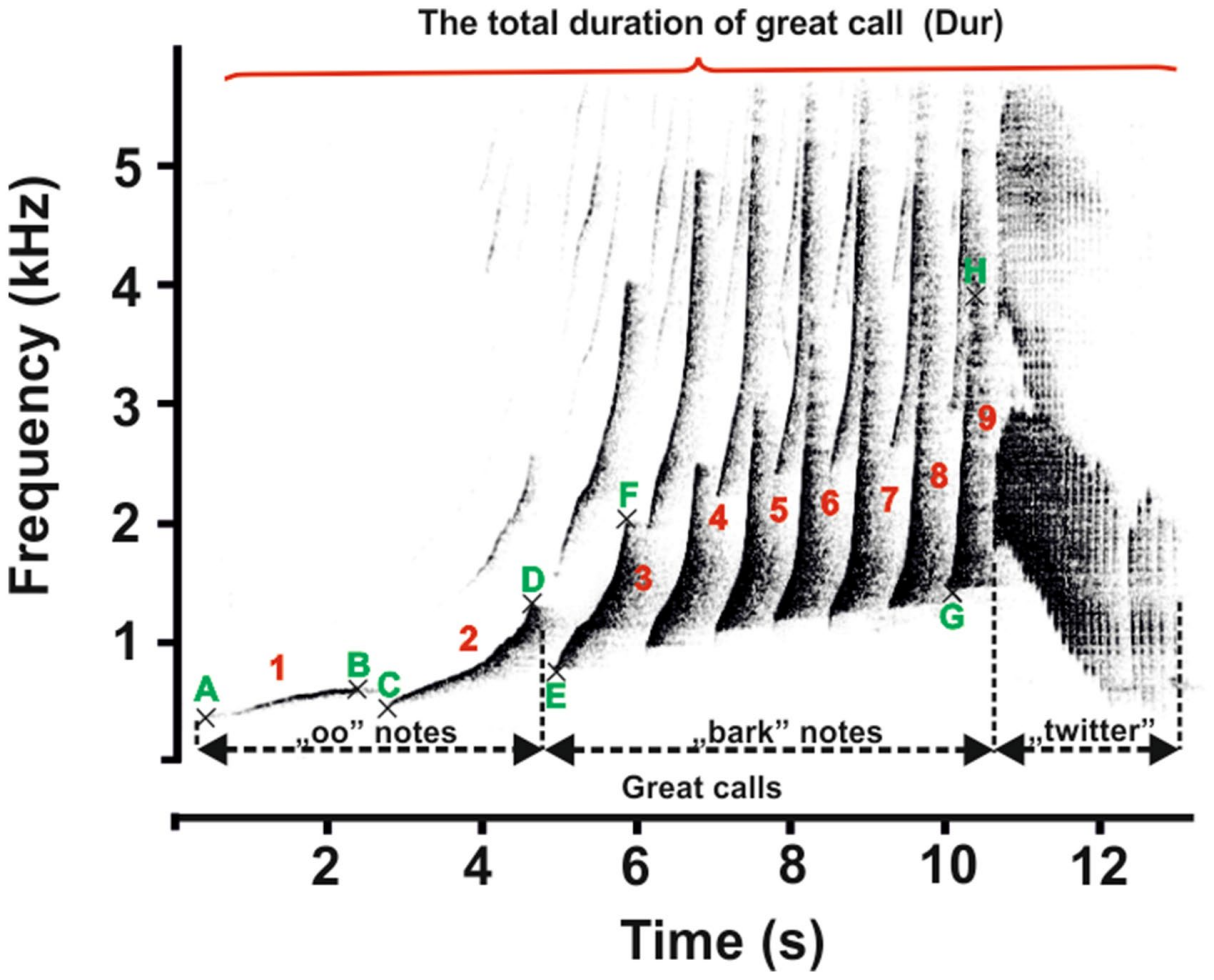
Representative spectrogram of the vocal patterns and acoustic parameters of the female southern yellow-cheeked gibbon’s great call. The red numbers indicate the number of notes (“oo” and “bark”); the green letters indicate the following: A and B – the minimum and maximum frequencies of the first “oo” notes; C and D – the minimum and maximum frequencies of the last “oo” notes; E and F – the minimum and maximum frequencies of the first “bark” notes; and A and B – the minimum and maximum frequencies of the last “bark” notes.

**Figure 4:**
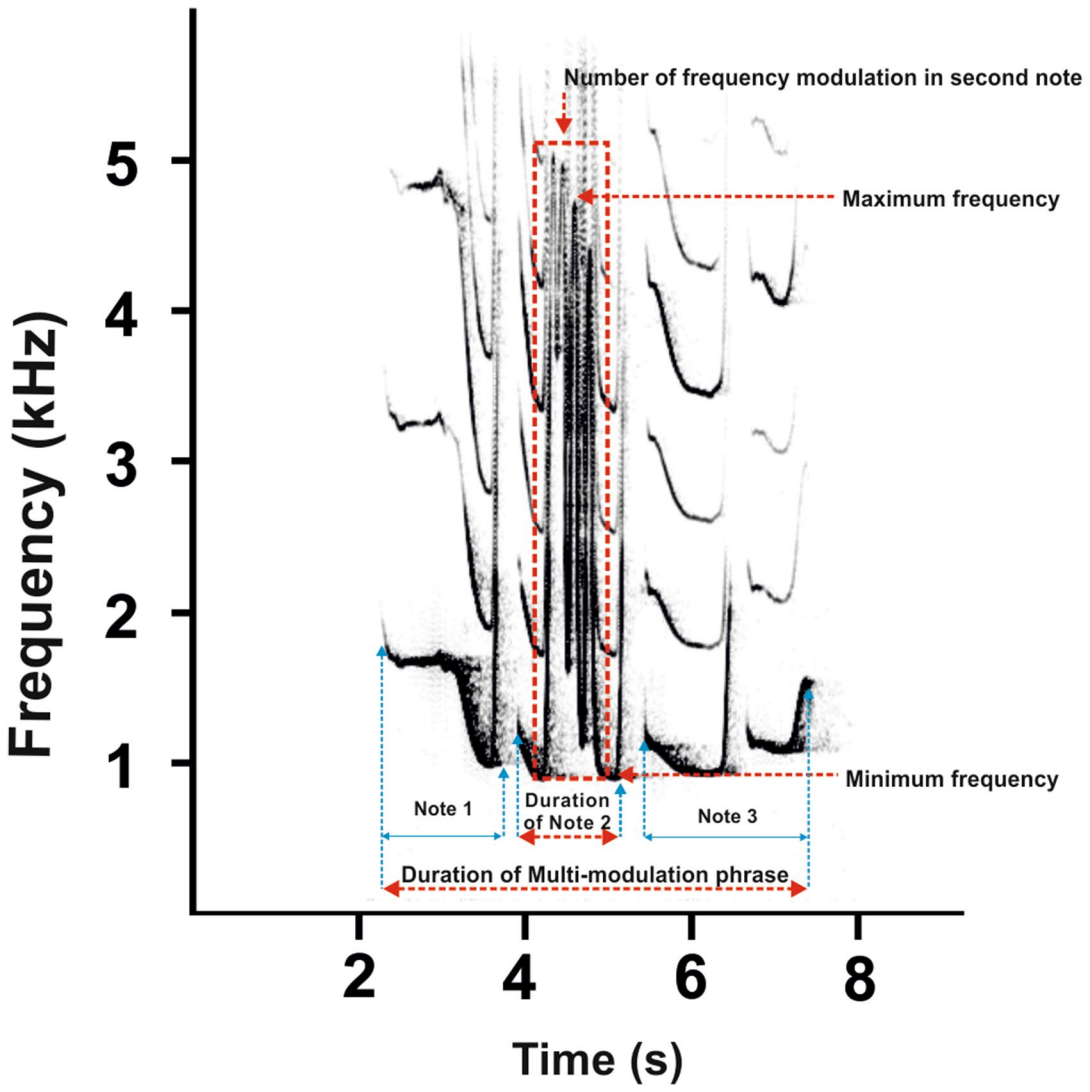
A representative spectrogram male call of southern yellow-cheeked gibbon displays [adapted from Hradec et al. (29)] divided into three notes. The most rapid change in frequency modulation is evident in the second note of the steep up-and-down sweeping sound (red dashed rectangle). Red dashed lines indicate the measured parameters of the multi-modulation phrase.

The recording devices (Marantz PMD 660 and M-Audio Micro Track II) were connected to a Rode NTG-2 semi-directional microphone, and all recordings were saved as waveform audio files. The sounds were recorded in mono in a 16-bit resolution and at a 44.1 kHz sampling rate. All recordings were saved as waveform audio files. The sampling frequency was reduced from 44.1 to 12 kHz for further analysis. Acoustic analysis was carried out using Avisoft SASLab Pro version 5.2 software (Avisoft Bioacoustics, Berlin, Germany). Spectrograms were generated under the following settings: FFT length = 1,024; frequency resolution = 12 Hz; temporal resolution = 21.3 ms; overlap = 75%; and window type = Hamming). The acoustic terminology used here for the characteristics of the great call and coda vocalization is in line with that proposed in previous studies (1, 4). The great call of “oo” notes comprises long notes that slowly increase in frequency by ≤2 kHz/s. Subsequent notes are short, with a steep frequency increase of >2 kHz/s. They are termed “bark” notes, making up the bark phase of the great call. After the acceleration peaks, the bark notes tail off into the twitter sound (Figure 3). Accordingly, we defined 11 temporal and spectral acoustic parameters corresponding to the great calls of adult females (Figure 3). Our measurement completely covered the temporal parameters, the duration of the great call (Dur), which included the “oo” and “bark” notes and twitter sounds. Moreover, the number of “oo” notes (Nsyloo) and “bark” notes (Nsylbark) were counted. The spectral parameters of these notes were measured as the minimum value of fundamental frequency (F0min, i.e., the minimum frequency for the “oo” and “bark” notes) and the maximum value of fundamental frequency (F0max, i.e., the maximum frequency for the “oo” and “bark” notes). The first and last “oo” and “bark” notes were measured to give an overall representation of the F0 distribution across the spectrum of the great call phrase (Figure 3, point A–H). The coda vocalizations are made up of three notes, i.e., Note 1, Note 2, and Last notes (Figure 4). The roll part of the second note of the multi-modulation phrase includes rapid frequency modulations, consisting of a steep up-and-down sweep (27, 29), which is more flexible than the overall pattern of the multi-modulation phrase. Therefore, our analysis of frequency parameters focused on this part. We measured the maximum value of fundamental frequency (kHz, Fmax) and the minimum value of fundamental frequency (kHz, Fmin). As for temporal parameters, we measured the total duration (s) of the coda vocalization (all three notes included, DurT) and the duration of roll part (DurR). Further, we counted the number of frequency modulations in the roll part of the second note (Number F).

### 2.4 Statistical analysis

All data were analyzed using R software, version 4.3.3 (33). Normal distribution was determined using the Kenward-Roger test, with an expected confidence level of 95%. The goodness of fit of each model (homoscedasticity, normality of errors, and independence) was tested by visual inspection of the residuals. The acoustic characteristics in females and males were highly inter-correlated. Therefore, we applied a principal component analysis (PCA) in the R software using the function *prcomp* (33). The PCA of the acoustic parameters resulted in three acoustic components (PCA 1–3), which explained 95% of the variation (Tables 2, 3). In females, the first principal component (Group 1 call component) included Freq A, Freq B, Freq C, Freq D, Freq E, Freq F and Nsylbark. The second principal component (Group 2 component) included Dur and Freq H. The third principal component (Group 3 component) included Nsyloo and Freq G. In males, the first principal component included DurR and Fmax. The second principal component (Group 2 call component) included Fmin and Number F. The third principal component (Group 3 call component) included DurT. Associations between acoustic parameters, animal age, and hormonal contraceptives were tested using multivariate Generalized Linear Mixed Models using the function *lmer* in package *lme4* (Bates et al., 2015). The model for females or males was designed so that the components (Group 1 call component, Group 2 component or Group 3 component) were considered as dependent variables. The period of hormonal contraceptive treatment (pre-, during- and post-treatment), modeled as interaction with each individual female or males, and the age of the female (10.8 to ⁓35 years) or males (12 to ~32 years) were both considered as fixed effects. The model was designed for repeated measurements with the individual female used as a random effect. A *p*-value ≤0.05 was considered statistically significant. The statistical differences between levels of effects were tested with using package *lsmenas* in R software (34).

**Table 2:** Characteristics of female variables included in the PCA.

Acoustic parameters	Description	Mean ± SD	PCA 1	PCA 2	PCA 3
Time interval between the start and end of great call (Total duration)	Dur	10.67 ± 1.36	−0.124309	0.786793	−0.052854
Total number of the oo notes	Nsyloo	2.06 ± 0.23	−0.203076	0.234995	−0.619777
Total number of the bark notes	Nsylbark	6.24 ± 1.47	−0.775175	0.230102	0.231896
Minimum value of fundamental frequency in the first oo notes	Freq A	475.21 ± 77.09	0.637328	−0.329296	0.080695
Maximum value of fundamental frequency in the first oo notes	Freq B	890.98 ± 297.80	0.833361	−0.039323	−0.056388
Minimum value of fundamental frequency in the last oo notes	Freq C	738.39 ± 174.97	0.881950	−0.032764	0.030327
Maximum value of fundamental frequency in the last oo notes	Freq D	1651.92 ± 315.48	0.860851	0.218714	0.007710
Minimum value of fundamental frequency in the first bark notes	Freq E	1024.67 ± 205.93	0.768300	0.346745	0.209305
Maximum value of fundamental frequency in the first bark notes	Freq F	2272.27 ± 315.26	0.731973	0.268323	−0.002177
Minimum value of fundamental frequency in the last bark notes	Freq G	1627.40 ± 187.17	−0.189103	−0.005313	0.746643
Maximum value of fundamental frequency in the last bark notes	Freq H	3820.71 ± 324.98	−0.073431	0.771608	0.116001

**Table 3:** Characteristics of male variables included in the PCA.

Acoustic parameters	Description	Mean ± SD	PCA 1	PCA 2	PCA 3
Time interval between the start and end of coda vocalization (Total duration)	DurT	5.21 ± 0.54	0.603958	0.220167	0.688930
Time interval in roll part of second note (Duration in roll part)	DurR	0.88 ± 0.26	0.708401	0.513194	0.059841
Maximum value of fundamental frequency	Fmax	4,954 ± 440	0.829886	−0.381055	−0.092599
Minimal value of fundamental frequency	Fmin	841 ± 351	−0.678865	0.528231	0.365299
Number of frequency modulation in roll part of second note	Number F	4.01 ± 0.67	0.240896	0.740201	−0.554764

## 3 Results

### 3.1 Great call

**Figure 5:**
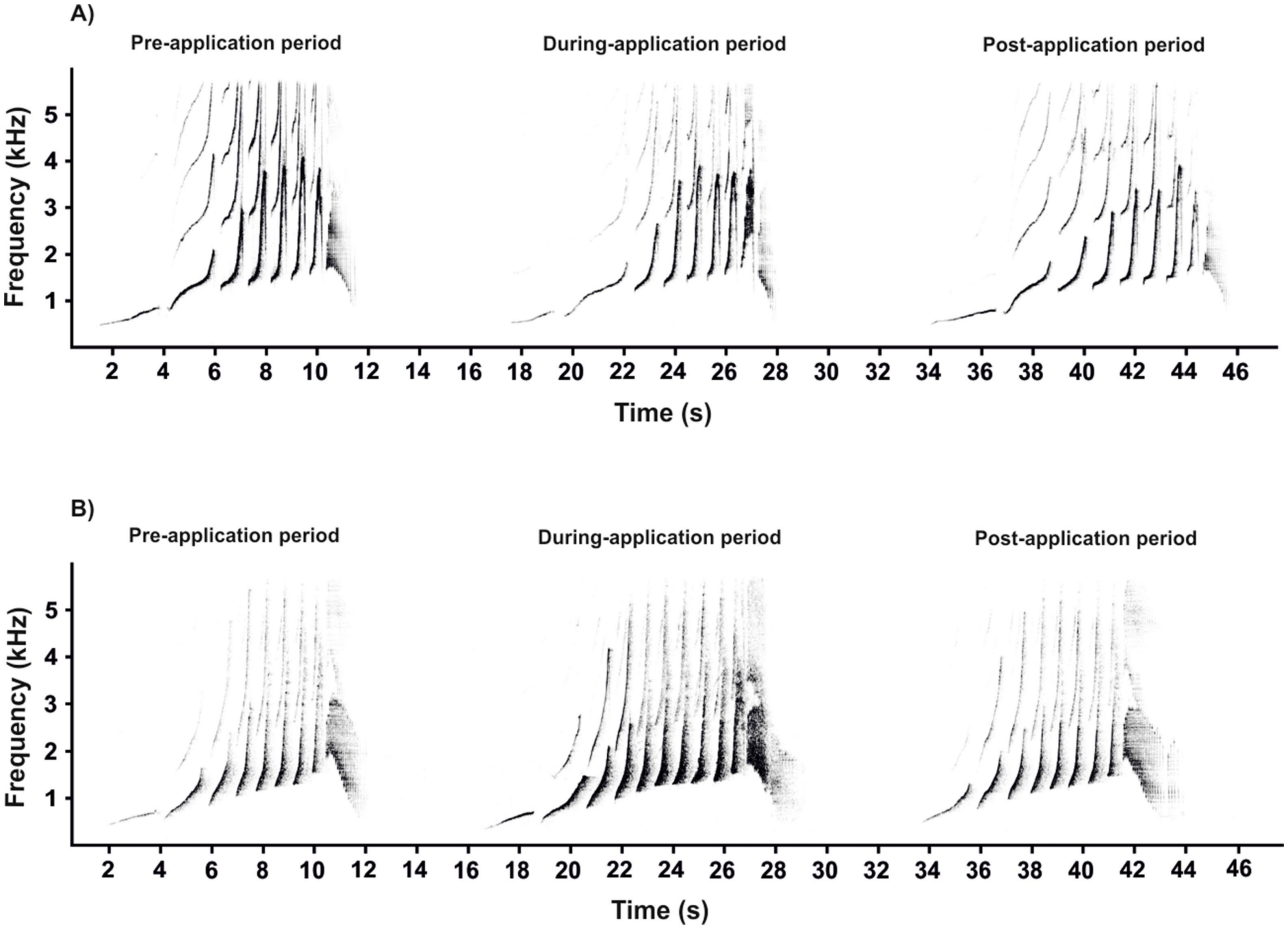
Representative spectrograms showing the structure of female great calls during three periods of the application of hormonal contraceptives: **(A)** Depo-Provera® and **(B)** Nexplanon®.

**Figure 6:**
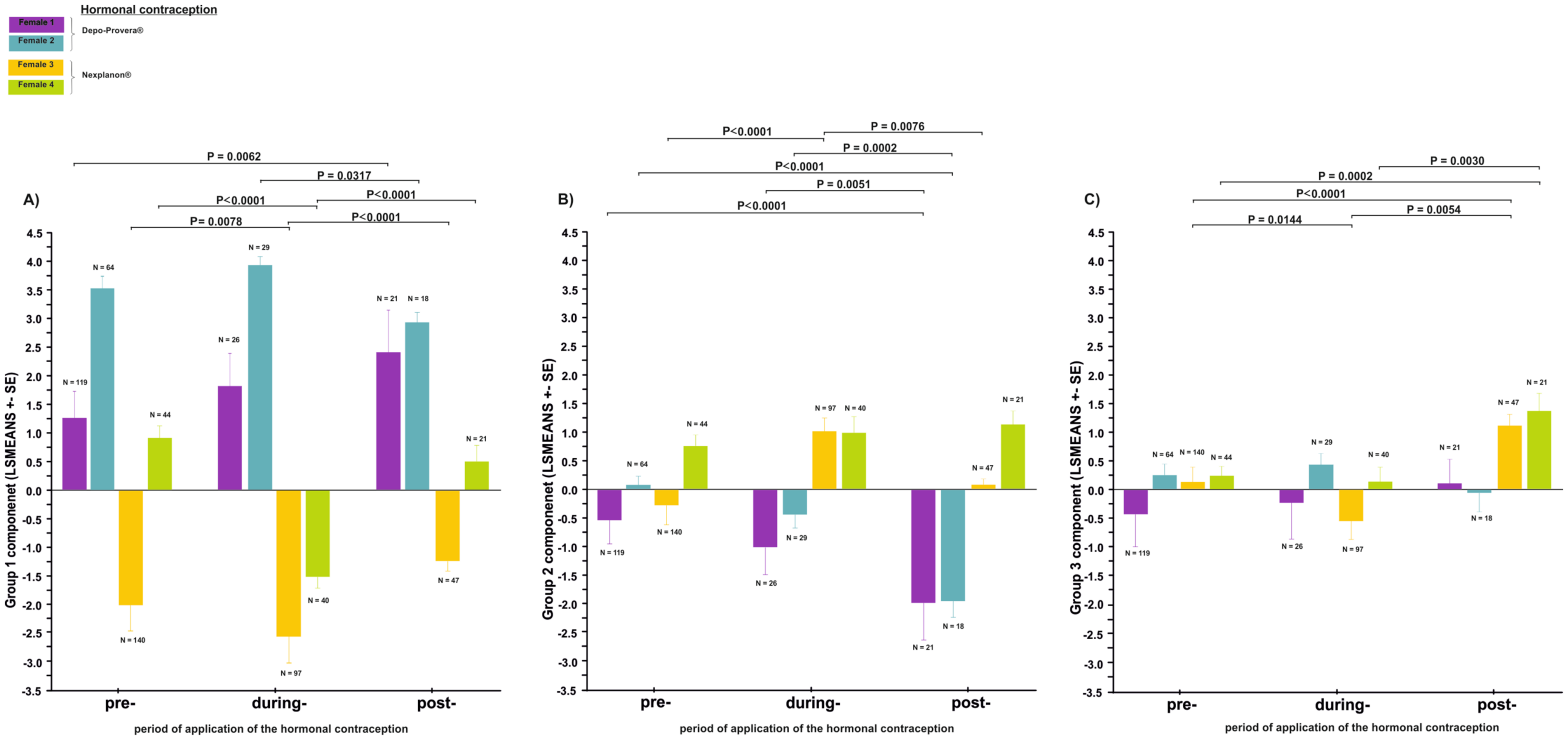
Differences in the acoustic parameters of the great call (least squares mean ± SE) during the three periods of hormonal contraceptive treatment (pre-treatment, during-treatment and post-treatment): **(A)** Group 1 call component, **(B)** Group 2 call component, **(C)** Group 3 call component.

We analyzed 666 great calls recorded during the three periods of contraceptive use (pre-, during- and post-application; Figures 5A,B; Supplementary Table S1). The Group 1 call component was affected by the contraceptive treatment period nested within individual females (Figure 6A). Application of the contraceptive Depo-Provera® did not result in any change in the value of the component. Once the effects of contraception wore off, in Female 1 the level of the component saw a significant increase (T = −3.111, *p* = 0.0062), but in Female 2 the component tended to return to pre-contraceptive levels. Conversely, the application of the contraceptive Nexplanon® resulted in a significant decrease in the value of the component (Female 3: T = 3.155, *p* = 0.0078; Female 4: T = 7.494, *p* < 0.0001). Once the effects of contraception wore off, in both females the value of the components saw a significant increase (Female 3: T = 4.247, *p* < 0.0001; Female 4: T = 4.805, *p* < 0.0001). These values exhibited a tendency to return to their pre-contraceptive levels.

The Group 2 call component was affected by the contraceptive treatment period nested within the individual females (Figure 6B). While the contraceptive Depo-Provera® was being administered, the level of the component decreased. Once the effects of contraception wore off, the levels of the component continued to decrease significantly in both females (Female 1: T = −3.188, *p* = 0.0051, Female 2: T = −4.169, *p* = 0.0002). In contrast, the level of the component in the during-application period of the contraceptive Nexplanon®, saw a significant increase in Female 3 (T = −6.299 *p* < 0.0001), but no change was observed in Female 4. After the contraceptive had worn off, a significant and sharp decrease was observed in Female 3 (T = −3.080, *p* = 0.0076), but no change was observed in Female 4.

The Group 3 call component was affected by the contraceptive treatment period nested within individual females (Figure 6C). The value of the group 3 component decreased due to the contraceptive (Nexplanon®); it increased sharply as the effect of the contraceptive wore off. This increase in the post-application period was clearly significant compared to both the pre-application (Female 3: T = −3.146, *p* = 0.0054; Female 4: T = −2.871, *p* = 0.0142) and during-application (Female 3: T = 5.110, p < 0.0001; Female 4: T = 3.444, *p* = 0.0030) periods.

### 3.2 Coda vocalization

**Figure 7:**
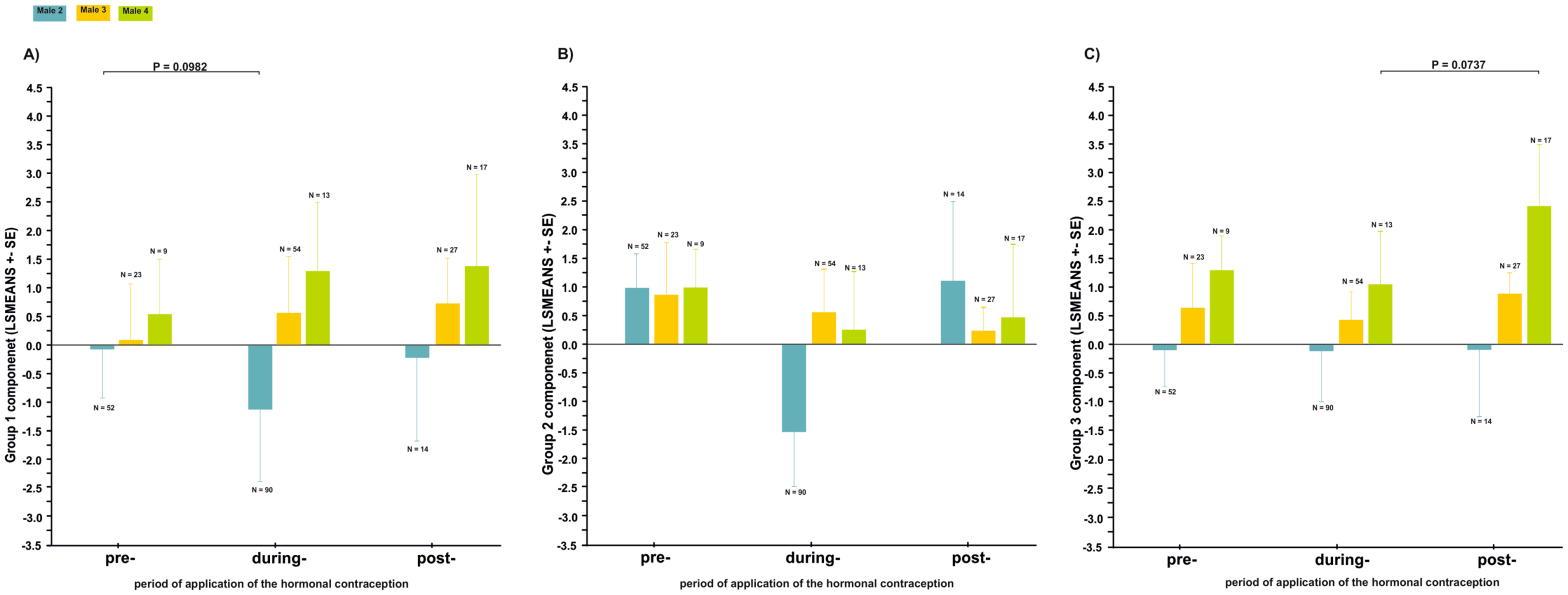
Differences in the acoustic parameters of the coda vocalization (least squares mean ± SE) in response to a change in the structure of a female’s great call during the three periods of hormonal contraceptive treatment (pre-treatment, during-treatment and post-treatment): **(A)** Group 1 call component, **(B)** Group 2 call component, **(C)** Group 3 call component.

We analyzed 299 coda vocalizations in males as a response to the females’ great call in the three periods of hormonal contraceptive treatment (i.e., pre-, during-, and post-treatment). No significant difference was demonstrated in any of the males’ call components (Group 1, Group 2 and Group 3) of the vocal structure during the three periods (Figure 7; Supplementary Table S2).

However, the Group 1 call component in the paired male 2 (T = −2.130; *p* = 0.0982, Figure 6A) and the Group 3 call component in the paired male 4 (T = 2.272; *p* = 0.0737, Figure 6C) showed a trend toward a shift in the structure of their coda vocalization over the three periods.

## 4 Discussion

Our study confirmed that the application of contraceptives affected the vocal pattern of the great call of female yellow-cheeked gibbons. We cannot confirm that paired males modified their coda vocalization directly in response to these changes in female singing. However, a trend toward such modification occurred in two cases.

### 4.1 Great call

We found that Group 1 and Group 2 components were most greatly affected by the application of the contraceptive Nexplanon® but not by the application of the contraceptive Depo-Provera® in the period during which the hormonal contraceptive was being used. Once the effects of the contraceptive had worn off (reversible phase, i.e., post-application of the hormonal contraceptive), the components affected by the contraceptive Depo-Provera® did not tend to return to pre-treatment values but continued to change. Conversely, components affected by the contraceptive Nexplanon® tended to return to pre-treatment values, although there was a difference in the size of the administered dose in both females (Table 1: 34 mg in Female 3; 68 mg in Female 4). We found a somewhat different pattern in the Group 3 component. In this case, the component values did not change after the application of the contraceptive Depo-Provera®. However, the change (an increase in the component) was noticeable when using the contraceptive Nexplanon®. Considering that our study is based on a relatively small sample size (four females) and that data collection was limited in time, the apparent impact of hormonal contraception revealed in the current study should be interpreted with caution.

As our findings show, the direction in which changes to the acoustic structure of the great call progressed differed between the two contraceptives. It is possible that these differences could be explained by medroxyprogesterone (Depo-Provera®) having more of an androgenic effect than etonogestrel (Nexplanon®), despite the fact that both types of contraceptives are based on synthetic progestins.

In a recent study by Maaskant and colleagues (35), it was shown that in female rhesus macaques (*Macaca mulatta*) and long-tailed macaques (*Macaca fascicularis*) a one-quarter and one-third dose of Nexplanon® was as effective as a whole dose (68 mg). These results are in agreement with our finding which indicates that half a dose (34 mg, Female 3) has approximately the same effect on the change in acoustic parameters as a whole dose (68 mg, Female 4). In contrast, the contraception Depo-Provera® was administered at the same dose (50 mg every 6 months) in both females. In the case of Female no. 1 (born ~1984), the change in the acoustic parameters of her great call could be related to age. However, our findings do not support such a conclusion because Group 1 components increased and Group 2 components decreased for both females equally, regardless of their different ages.

### 4.2 Coda vocalization

Our analysis focused mainly on the second note (i.e., roll part) of the coda vocalization in paired males of southern yellow-cheeked gibbons, which is more flexible than the overall pattern (29, 36). Our records come from three males from three different zoological parks and are based on a relatively small sample size (approximately half of the data was not suitable for acoustic analysis), suggesting that these results should be interpreted with caution. This small sample size probably explains why our findings suggest that neither the temporal nor spectral acoustic parameters of the roll part of coda vocalization was affected in direct response to a change in the structure of the females’ great call across three periods of hormonal contraceptive treatment (i.e., pre-, during- and post-treatment).

That said, in two cases there appears to be a tendency for the males’ vocal pattern to change in response to a change in the structure of the females’ great call during the observation periods. A recent study by Terleph and colleagues (30) revealed that paired males of white-handed gibbons (*Hylobates lar*) rapidly and flexibly adjust their coda vocalization in direct response to a female’s singing changes. Whether this ability is also present in paired male southern yellow-cheeked gibbons is still an open question that requires further investigation.

### 4.3 Population management for the captive southern yellow-cheeked gibbon

The EAZA’s population of captive southern yellow-cheeked gibbons—numbering around 99 individuals—is one of the largest in the world (37) and could play a key role in conserving the species for future generations. In many zoos, hormonal contraception is the most widely used form of population management, mainly because of its long-term positive results in preventing pregnancy (5). However, the use of hormonal contraception should be carefully considered, given the biology of this species. In most captive and wild gibbon species, mating individuals combine their respective songs in well-coordinated duets or solo songs, including the female’s great call—possibly emphasizing their role in territorial defense and/or the maintenance of pair and family bonds (38–40). Therefore, further research is needed to determine whether hormonal contraception (one type of hormonal contraceptive administered at a constant dosage) alters not only the vocal pattern but also the behavior of female gibbons.

In the absence of other comparable studies, we can only speculate about the negative effect of contraception on the welfare of both female and male gibbons. One interesting finding comes from Karaskiewicz et al. (21) whose study of captive coppery titi monkeys (*Plecturocebus cupreus*)—like gibbons, also pair-living primates performing well-coordinated duets—revealed that no significant differences in affiliative behaviors were observed between contraceptive-treated and untreated pairs. Other research on hormonal contraception in female non-human primates, including that of baboons (*Papio* sp.) and macaques (*Macaca* spp.), however, has shown mixed effects on behavior and hence animal welfare (18, 19, 41–43). For example, in a study by Crawford and colleagues (17), hormonal contraceptives are reported has having disrupted social hierarchy in ring-tailed lemurs (*Lemur catta*) by altering olfactory stimuli in females and potentially disrupting mate choice and kin recognition. Although our study is limited since it relies on the observation of a small sample size (four females) and the fact that data on contraceptive use were collected as part of zoos’ standard procedure, e.g., monitoring the effects of two types of hormonal contraception and different doses of the contraception Nexplanon®.

The findings in ring-tailed lemurs, together with our own results, therefore, point to the need for a comprehensive analysis of the potential impact of contraception on animal welfare, i.e., including all behavioral patterns of an individual.

## 5 Conclusion

Our study is the first of its kind to provide evidence that changes occur in the spectral and temporal parameters of the great call (produced by adult female southern yellow-cheeked gibbons) during the three periods of hormonal contraceptive treatment (pre-, during- and post-treatment). In contrast, our study did not find that paired males adjust their coda vocalization in direct response to a female’s singing changes. However, these results should only be considered as preliminary evidence, as more research is needed. Future studies should include larger sample size, the effects of behavior, and different forms of contraception—as the effects of different hormonal treatments may vary. Data obtained from such studies could help us provide an optimal solution for reproductive management of gibbons and ensure that the welfare standards are met.

## Data Availability

The raw data supporting the conclusions of this article will be made available by the authors, without undue reservation.
