# Supplementary material for: Effects of hormonal contraception on vocal patterns of captive southern yellow-cheeked gibbons (Nomascus gabriellae)
Source: Front Vet Sci. 2025 Apr 7;12:1574926. doi: 10.3389/fvets.2025.1574926 (PMC12009915; doi:10.3389/fvets.2025.1574926)
Supplement: Supplementary file 1 [file Table_1.docx]

***Supplementary Material***

**Table S1:** The relationship the three principal component analysis (PCA 1-3) in the three periods of hormonal contraceptive treatment (pre-treatment, during treatment and post-treatment) in four females.

| **Adult females** | **PCA 1** | | | **PCA 2** | | | **PCA 3** | | |
| --- | --- | --- | --- | --- | --- | --- | --- | --- | --- |
|  | **Period of data collection** | **Estimate±SD** | **P-value** | **Period of data collection** | **Estimate±SD** | **P-value** | **Period of data collection** | **Estimate±SD** | **P-value** |
| **Female 1** | pre - post | -1.143 ± 0.367 | **0.0062** | pre - post | 1.730 ± 0.332 | **<.0001** | pre - post | -0.499 ± 0.365 | 0.3600 |
|  | pre - during | -0.442 ± 0.264 | 0.2206 | pre - during | 0.574 ± 0.249 | 0.0607 | pre - during | -0.216 ± 0.258 | 0.6803 |
|  | post - during | 0.701 ± 0.398 | 0.1861 | post - during | -1.156 ± 0.363 | **0.0051** | post - during | 0.283 ± 0.394 | 0.7537 |
| **Female 2** | pre - post | 0.619 ± 0.389 | 0.2536 | pre - post | 1.934 ± 0.355 | **<.0001** | pre - post | 0.351 ± 0.384 | 0.6330 |
|  | pre - during | -0.378 ± 0.300 | 0.4229 | pre - during | 0.424 ± 0.280 | 0.2900 | pre - during | -0.063 ± 0.294 | 0.9752 |
|  | post - during | -0.996 ± 0.389 | **0.0317** | post - during | -1.510 ± 0.362 | **0.0002** | post - during | -0.414 ± 0.381 | 0.5253 |
| **Female 3** | pre - post | -0.758 ± 0.347 | 0.0760 | pre - post | -0.334 ± 0.311 | 0.5321 | pre - post | -1.085 ± 0.345 | **0.0054** |
|  | pre - during | 0.650 ± 0.206 | **0.0078** | pre - during | -1.287 ± 0.204 | **<.0001** | pre - during | 0.579 ± 0.198 | **0.0144** |
|  | post - during | 1.408 ± 0.332 | **0.0001** | post - during | -0.953 ± 0.309 | **0.0076** | post - during | 1.665 ± 0.326 | **<.0001** |
| **Female 4** | pre - post | 0.441 ± 0.423 | 0.5518 | pre - post | -0.367 ± 0.401 | 0.6323 | pre - post | -1.186 ± 0.413 | **0.0142** |
|  | pre - during | 2.324 ± 0.310 | **<.0001** | pre - during | -0.251 ± 0.301 | 0.6840 | pre - during | 0.120 ± 0.301 | 0.9156 |
|  | post - during | 1.883 0.392 | **<.0001** | post - during | 0.116 ± 0.382 | 0.9503 | post - during | 1.306 ± 0.379 | **0.0030** |
