# Supplementary material for: Effects of hormonal contraception on vocal patterns of captive southern yellow-cheeked gibbons (Nomascus gabriellae)
Source: Front Vet Sci. 2025 Apr 7;12:1574926. doi: 10.3389/fvets.2025.1574926 (PMC12009915; doi:10.3389/fvets.2025.1574926)
Supplement: Supplementary file 2 [file Table_2.docx]

***Supplementary Material***

**Table S2:** The relationship the three principal components analysis (PCA 1-3) coda vocalizations in three males as a response to female´s great call in the three periods of hormonal contraceptive treatment (pre-treatment, during treatment and post-treatment).

| **Adult males** | **PCA 1** | | | **PCA 2** | | | **PCA 3** | | |
| --- | --- | --- | --- | --- | --- | --- | --- | --- | --- |
|  | **Period of data collection** | **Estimate±SD** | **P-value** | **Period of data collection** | **Estimate±SD** | **P-value** | **Period of data collection** | **Estimate±SD** | **P-value** |
| **Male 2** | pre - post | -0.157 ± 0.887 | 0.9829 | pre - post | -0.209 ± 0.748 | 0.8376 | pre - post | -0.0108 ± 0.668 | 0.9999 |
|  | pre - during | -1.156 ± 0.544 | 0.0982 | pre - during | -0.564 ± 0.460 | 0.4500 | pre - during | -0.031 ± 0.423 | 0.9970 |
|  | post - during | -1.001 ± 0.737 | 0.3717 | post - during | -0.355 ± 0.624 | 0.8376 | post - during | -0.020 ± 0.564 | 0.9993 |
| **Male 3** | pre - post | -0.786 ± 0.874 | 0.6419 | pre - post | -0.661 ± 0.730 | 0.6387 | pre - post | 0.192 ± 0.632 | 0.9506 |
|  | pre - during | -0.614 ± 0.365 | 0.2304 | pre - during | -0.348 ± 0.313 | 0.5132 | pre - during | -0.2728 ± 0.292 | 0.6229 |
|  | post - during | 0.173 ± 0.791 | 0.9740 | post - during | 0.312 ± 0.665 | 0.8855 | post - during | -0.464 ± 0.588 | 0.7107 |
| **Male 4** | pre - post | 0.798 ± 1.030 | 0.7196 | pre - post | 0.558 ± 0.869 | 0.7974 | pre - post | -1.138 ± 0.779 | 0.3191 |
|  | pre - during | 0.727 ± 0.699 | 0.5571 | pre - during | 1.199 ± 0.595 | 0.1248 | pre - during | 0.169 ± 0.550 | 0.9488 |
|  | post - during | -0.071 ± 0.735 | 0.9948 | post - during | 0.640 ± 1.023 | 0.5676 | post - during | 1.308 ± 0.576 | 0.0737 |
